# MKL1 regulates hepatocellular carcinoma cell proliferation, migration and apoptosis via the COMPASS complex and NF-κB signaling

**DOI:** 10.1186/s12885-021-08185-w

**Published:** 2021-11-06

**Authors:** Zhao Liu, Jiuzheng Sun, Chuanzhi Li, Liyou Xu, Jun Liu

**Affiliations:** 1 Department of Hepatobiliary and Pancreatic Surgery, Jinan Central Hospital, Cheeloo College of Medicine, Shandong University, Jinan, China; 2 Department of Liver Transplantation and Hepatobiliary Surgery, Shandong Provincial Hospital, Cheeloo College of Medicine, Shandong University, Jinan, China; 3 Department of Liver Transplantation and Hepatobiliary Surgery, Shandong Provincial Hospital Affiliated to Shandong First Medical University, Jinan, China

**Keywords:** Ash2, Hepatocellular carcinoma, MKL1, p65, Wdr5

## Abstract

**Background:**

Histone modification plays essential roles in hepatocellular carcinoma (HCC) pathogenesis, but the regulatory mechanisms remain poorly understood. In this study, we aimed to analyze the roles of Megakaryoblastic leukemia 1 (MKL1) and its regulation of COMPASS (complex of proteins associated with Set1) in HCC cells.

**Methods:**

MKL1 expression in clinical tissues and cell lines were detected by bioinformatics, qRT-PCR and western blot. MKL1 expression in HCC cells were silenced with siRNA, followed by cell proliferation evaluation via Edu staining and colony formation, migration and invasion using the Transwell system, and apoptosis by Hoechst staining. HCC cell tumorigenesis was assessed by cancer cell line-based xenograft model, combined with H&E staining and IHC assays.

**Results:**

MKL1 expression was elevated in HCC cells and clinical tissues which was correlated with poor prognosis. MKL1 silencing significantly repressed proliferation, migration, invasion and colony formation but enhanced apoptosis in HepG2 and Huh-7 cells. MKL1 silencing also inhibited COMPASS components and p65 protein expression in HepG2 and Huh-7 cells. HepG2 cell tumorigenesis in nude mice was severely impaired by MKL1 knockdown, resulted into suppressed Ki67 expression and cell proliferation.

**Conclusion:**

MKL1 promotes HCC pathogenesis by regulating hepatic cell proliferation, migration and apoptosis via the COMPASS complex and NF-κB signaling.

**Supplementary Information:**

The online version contains supplementary material available at 10.1186/s12885-021-08185-w.

## Background

Hepatocellular carcinoma (HCC) is still one common human malignancy featured with high incidence and mortality, which also is the major liver cancer subtype and accounted for more than 82% cancer cases originated in the haptic tissues all over the world [1–3]. At present, clinical treatment of HCC are mainly dependent on the appropriate combination of surgical resection, chemotherapy, liver transplantation and even targeted therapies, according the individual pathophysiological features [4, 5]. Unfortunately, the incidences of hepatocellular carcinoma have been continuously increasing during the past decades, especially in Asian countries like China, Mongolia, Southeast Asia areas and Western and Eastern Africa [1, 6]. The limited efficacies of recent therapeutic regiments have been possibly attributed to the severity of cirrhosis symptoms, high rates of multiple drug resistances, liver organ donor shortage and diversity of histological properties [4–6]. Extensive epidemiological investigations demonstrated that the HCC incidence has been closely linked with various causative factors such as chronic infections induced by hepatitis B and hepatitis C viruses, alcohol assumption, hepatic cirrhosis, as well as other hepatic pathogenic conditions such as NASH (nonalcoholic steatohepatitis) and NAFLD (nonalcoholic fatty liver diseases) [6, 7]. However, the pathogenic mechanisms driving hepatocellular carcinoma initiation and progression still remains poorly understood.

The molecular pathogenesis of HCC was known as a multi-step process during which cellular and molecular alterations were progressively accumulated, such as various genomic mutations, epigenetic regulations, and alterations in transcriptome, cellular signaling pathways and metabolic processes [8, 9]. Among them, focal implications of several oncogenes like c-Met proto-oncogene (MET) and telomerase reverse transcriptase (TERT), as well as the genetic deletions of tumor suppressor genes including TP53 (Tumor Protein P53) and PTEN (phosphatase and tensin homolog) were characterized as essential molecular events underlying HCC development and progression [8, 10–12]. Also, HCC initiation, development and metastasis have been previously reported to be mediated by significant changes in pleiotropic cellular signaling pathways towards enhanced cell proliferation, migration and invasion, repressed hepatic cell apoptosis and also chemotherapy resistance [8, 9]. For instance, the metastasis of hepatocellular carcinoma cells could be enhanced by the nuclear factor kappa B (NF-kB) signaling and resultant high expression of matrix metalloproteinases 2 and 9 (MMP2/9) in responses to interleukin 17A [13]. Moreover, the activation of NF-kB signaling pathway contributed to the development of resistance to sorafenib resistance by promoting the expression of CD47 in hepatocellular carcinoma tissues [14]. Nevertheless, the molecular landscape underlying HCC pathogenesis such as mechanisms of NF-kB signaling regulations are far from being fully elucidated.

Epigenetic machineries have been recently characterized as essential regulator of cellular processes and signaling pathways driving cancer development and progression [15–18]. Importantly, the pathogenic processes of chronic liver diseases, fibrosis/cirrhosis and hepatocellular carcinoma were also critically regulated by extensive epigenetic controls, mediated by DNA methylation, histone modifications as well as several types of non-coding RNA molecules [19, 20]. Recent discoveries in the liver epigenetics field have been suggested as new targets for HCC diagnosis and treatment due to the high potentials of reverse epigenetic changes and epigenome reprograming [20]. For instance, the formation of H3K4 trimethylation could be catalyzed by the COMPASS (complex of proteins associated with Set1) complex, composed of ASH2 (absent, small or homeotic discs 2), SET1 (SET-domain-containing protein 1) and WDR5 (WD Repeat-containing Protein 5), which could regulates the downstream NF-κB signaling activation [21]. The elevated levels of trimethylation at histone H3 lysine 4 (H3K4), which was established as one key epigenetic mechanism enhancing target gene transcription, was positively correlated with poor prognosis of hepatocellular carcinoma patients [22]. Furthermore, Megakaryoblastic leukemia 1 (MKL1), alternatively known as MRTF-A (myocardin-related transcription factor A), regulates endothelial-to-mesenchymal transition and oncogene-induced senescence during pathogenesis of hepatocellular carcinoma and other liver disorders [23, 24]. It has been found that MKL1 is overexpressed in hepatocellular carcinoma and plays an important role in the growth of hepatocellular carcinoma and the aging-induced by oncogenes, and as a new target for the treatment of human liver cancer [25]. MKL1 also interacts with the COMPASS complex to promote the colitis development through regulating NF-κB nuclear enrichment and the accumulation of H3K4 trimethylation [21]. However, whether the MKL1/COMPASS/NF-kB axis mediated HCC development remains unclear.

In this study, we aimed to investigate the expressional alterations and pathogenic roles of MKL1 gene in hepatocellular carcinoma pathogenesis using both cellular and mouse models, as well as the involvements of downstream COMPASS complex and NF-κB signaling pathways. These analyses would provide novel insights into the changes of epigenetic landscapes associated with HCC initiation and development, which could also be further explored for liver cancer diagnosis and targeted therapy.

## Methods

### Cell culture

The human hepatocellular carcinoma cell lines HepG2 (CL-0103, procell), Hep 3B (CL-0101, procell) and Huh-7 CL-0120, procell) were purchased from the American Type Culture Collection (procell; China). Above cell lines were cultured in the Dulbecco’s modified Eagle medium (DMEM) which was supplemented with 10% FBS (fetal bovine serum; Thermo Fishier Scientific) and 100 U/mL penicillin/streptomycin (Sigma Aldrich) at 37 °C in a humidified thermal incubator with 5% CO_2_. The identities of cultured cells were confirmed by the STR (short tandem repeat) profiling method.

### Quantitative RT-PCR

Relative mRNA levels in cultured cells or mouse liver tissues were measured by the quantitative RT-PCR (qRT-PCR) method. Briefly, total RNA samples were extracted form hepatocellular carcinoma cell lines or mouse hepatic tissues using the Trizol kit (#15596026; Thermo Fishier Scientific) strictly following the manufacturer’s instructions. The concentrations of extracted RNA samples were measured using the Thermo Scientific NanoDrop™ 1000 spectrophotometer. Subsequently, the cDNA library from about 2 μg RNA per group was then established by RT-PCR method using the High-capacity RT Kit (#4368814; Thermo Fisher Scientific) according to the protocol provided by the manufacturer. Gene expression levels were then detected by quantitative PCR assay using the SYBR Green qPCR Master mix kit (#330500; Thermo Fishier Scientific) following the producer’s instructions, which was finished by pre-denaturation at 95 °C for 90 s, followed by 41 rounds of denaturation for at 94 °C for 15 s, annealing at 58 °C for 21 s and elongation at 72 °C for 24 s. The relative expressional levels were finally calculated via the standard 2^-△△Ct^ method, by calibration to the expression of GAPDH (Glyceraldehyde-3-phosphate dehydrogenase). Primers used in mRNA quantitation were shown in Table 1.

**Table 1:** Primers used for mRNA quantitation by qRT-PCR

Primer ID	Primer sequences (5′-3′)	Product length (bp)
MKL1-F	CAAACGGAAGATTCGTTCCCG	135
MKL1-R	TTGAGGTCATCGGCTAGTCTG	
P65-1F	CTTCCAAGAAGAGCAGCGTG	183
P65-1R	CCAGAGTTTCGGTTCACTCG	
Set1-F	TTGCCATGTCAGGTCCAAAAA	161
Set1-R	CGTACTTACGGCACATATCCTTC	
Wdr5-F	GCTGCAACTTCAATCCCCAGT	88
Wdr5-R	CACTTCCCTGTTTTCACATCCC	
Ash2-F	AGGGACCACCCCGGTC	165
Ash2-R	CAGCTGGAGAAGTCGAGTAG	
GAPDH F	TGTTCGTCATGGGTGTGAAC	154
GAPDH R	ATGGCATGGACTGTGGTCAT	

### Western botting

The extraction of total proteins from cultured hepatocellular carcinoma cell lines or mouse liver tissues were done using the Total Protein Extraction kit (#C510003; Sangon Biotech, Shanghai, China) following producer’s protocol. After protein concentration determination by BCA method, about 30 μg protein of each group were boiled at 100 °C for 5 min in protein loading buffer, separated by 10–12% SDS-PAGE, and blotted onto PVDF membranes (Millipore). The PVDF membranes containing protein samples were blocked with lipid-free milk solution (5%) for 1.5 h at room temperature, incubated with diluted primary and secondary antibodies, which was finally developed using the Highly sensitive ECL (enhanced chemiluminescence) luminescence reagent (#C500044; Sangon Biotech, Shanghai, China). The abundances of GAPDH proteins were simultaneously detected as the internal standard. Primary antibodies used for quantitation included anti-MKL1 (#ab49311; 1: 6000; Abcam), anti-P65 (#8242; 1: 5000; CST), anti-SET1 (#61702; 1: 4000; CST), anti-WDR5 (#13105; 1: 5000; CST), anti-ASH2 (#5019; 1: 2000; CST), anti-GAPDH (#ab181602; 1: 5000; Abcam), anti-Bcl-2 (#3498; 1:1000; CST),anti-Bax(#5023; 1:1000;CST), and anti-Cleaved-caspase-3(#9654; 1:1000; CST) .

### Cell transfection

To knockdown MKL1 gene expression in hepatocellular carcinoma cells, three siRNAs targeting LRG1 including siMIL1-I (5′-GCUGAAGAGAGCCAGACUATT-3′), siMIL1-II (5′-GCCUGAAGGAAGCCAUCAUTT-3′), siMIL1-III (5′-CCAAGGAGCUGAAGCCAAATT-3′) and the negative control (NC) sequence (5′-UUCUCCGAACGUGUCACGUTT-3′) were synthesized (Sangon Biotech, Shanghai, China) and transfected into HepG2 cells using the Lipofectamine 3000 reagent (#L3000001; Thermo Fishier Scientific) according to the producer’s instructions. The above sequence with the high efficiency of gene expression silencing was then selected as the Small hairpin RNA (shMKL1) and ligated with the lenti-virus LV003 vector and the negative control sequence was used as the Scramble (shSc) sequence. Above vectors were then used for transfections of the HEK293T cells together with packaging vectors for preparation of recombinant virus vectors, which were then transfected into the HepG2 cells as introduced above.

### Edu staining

The proliferation of HepG2 and HuH-7 cells were detected by the Edu staining method using the EdU Staining Kit (#ab219801; Abcam) according to the producer’s instructions. Briefly, cultured liver cancer cells were seeded in 96-well plate and culture overnight until the cell confluency reach about 65%, which were then incubated with 10 μM EdU for 3 h at 37 °C, mixed with 1X Fixative Solution and incubated at room temperature for 12 min in darkness. Subsequently, cells were incubated with 1X Permeabilization Buffer for 25 min at room temperature, incubated with 90 μL Reaction mix for 25 min, incubated with DAPI solution for 15 min in darkness, and finally observed with laser confocal microscope (Olympus, Japan).

### Cell migration and invasion

The migration and invasion capacities of HepG2 and HuH-7 cells were evaluated using the Transwell system (Corning, USA). For detection of cancer cell migration, the upper chambers of Transwell system were filled with serum-free DMEM medium with penicillin/streptomycin (1%), while the low chambers were full of complete DMEM medium containing FBS (10%) and penicillin/streptomycin (1%). Cancer cells were seeded on the upper chamber, incubated under normal cell culture conditions for 2 days, and liver cancer cells migrated into the lower chambers were counted and photographed under microscopy after being stained with 0.1% crystal violet for 10 min. To assess cell invasion, liver cancer cells were analyzed following the same procedures, except for that the inner sides of the Transwell plates were first coated with Matrigel (BD Biosciences).

### Colony formation

The proliferation of HepG2 and HuH-7 cells were also analyzed by the colony formation assay as follows. Cultured HepG2 and HuH-7 cells were collected by centrifuge at 800 g for 5 min, resuspended in DMEM medium and seeded in seeded at 6-well plates under the density of approximately 150 cells per well. After being cultured under normal conditions for 12–14 days, the cell colonies formed in 6-well plates were fixed with 4% paraformaldehyde for 15 min and stained at room temperature with 0.1% crystal violet solution for 20 min. The formation of cell colonies was defined by the aggregation of over 50 cells together.

### Cell apoptosis

HepG2 and HuH-7 cells were first stained with 5 μg/ml Hoechst 33258 (#H1398; Thermo Fishier Scientific) for 15 min at room temperature in darkness, which were then washed with 1 mL PBS solution for three times and resuspended in PBS followed by observation under fluorescence microscopy. The apoptosis of HepG2 and HuH-7 cells were finally evaluated based on at least three biological replicates.

### In vivo tumorigenesis

The tumorigenesis capacities of HepG2 cells were analyzed by the cancer cell line-based xenograft (CDX) model. Briefly, approximately female BALB/c nude mice aged 5 weeks were purchased from the Experimental Animal Center of the Shandong University and maintained in standard pathogen-free environment. Approximately 2 × 10^6^ HepG2 resuspended in PBS solution were injected subcutaneously into the BALB/c nude mice. After being sustained under normal conditions for 1 month, the mice were anaesthetized by injection of sodium pentobarbital (125 mg/kg) and sacrificed by carbon dioxide asphyxiation, and subcutaneous tumors were collected surgically for analysis of tumor volumes and weights. All operation on nude mice were pre-approved by the Animal Care and Use Committee of the Shandong University.

### Histological evaluation

The histological evaluation of tumor implanted tumors in nude mice were finished by combination of hematoxylin and eosin (H&E) staining and IHC (immunohistochemistry) assays. Briefly, tumor tissue sections (5 μm thick) were fixed with 4% neutral phosphate-buffered formalin at 4 °C, embedded with paraffin, and subjected to staining using the Hematoxylin-Eosin staining kit (#E607318; Sangon Biotech, Shanghai, China) according to the manufacturer’s instructions. The expression of Ki67 proteins in the tumor tissues were also detected by the Ki67 Cell Proliferation Kit (IHC) (#E607235; Sangon Biotech, Shanghai, China) following the producer’s instructions. The tissue slices were taken by a microscope (Olympus, Japan).

### Bioinformatics and statistical analysis

The expression of MKL1 gene in clinical tissues and its correlation with survival time of HCC patients were analyzed using the Oncomine and the human protein atlas databases respectively. The quantitative data presented as mean ± standard deviation were analyzed using the SPSS 20.0 software. Differences between two or more groups were evaluated by the student’s T test or ANOVA (analysis of variance). A *P* value of < 0.05 was used to define significant differences.

## Results

### Elevated expression of MKL1 gene in hepatocellular carcinoma

**Fig. 1:**
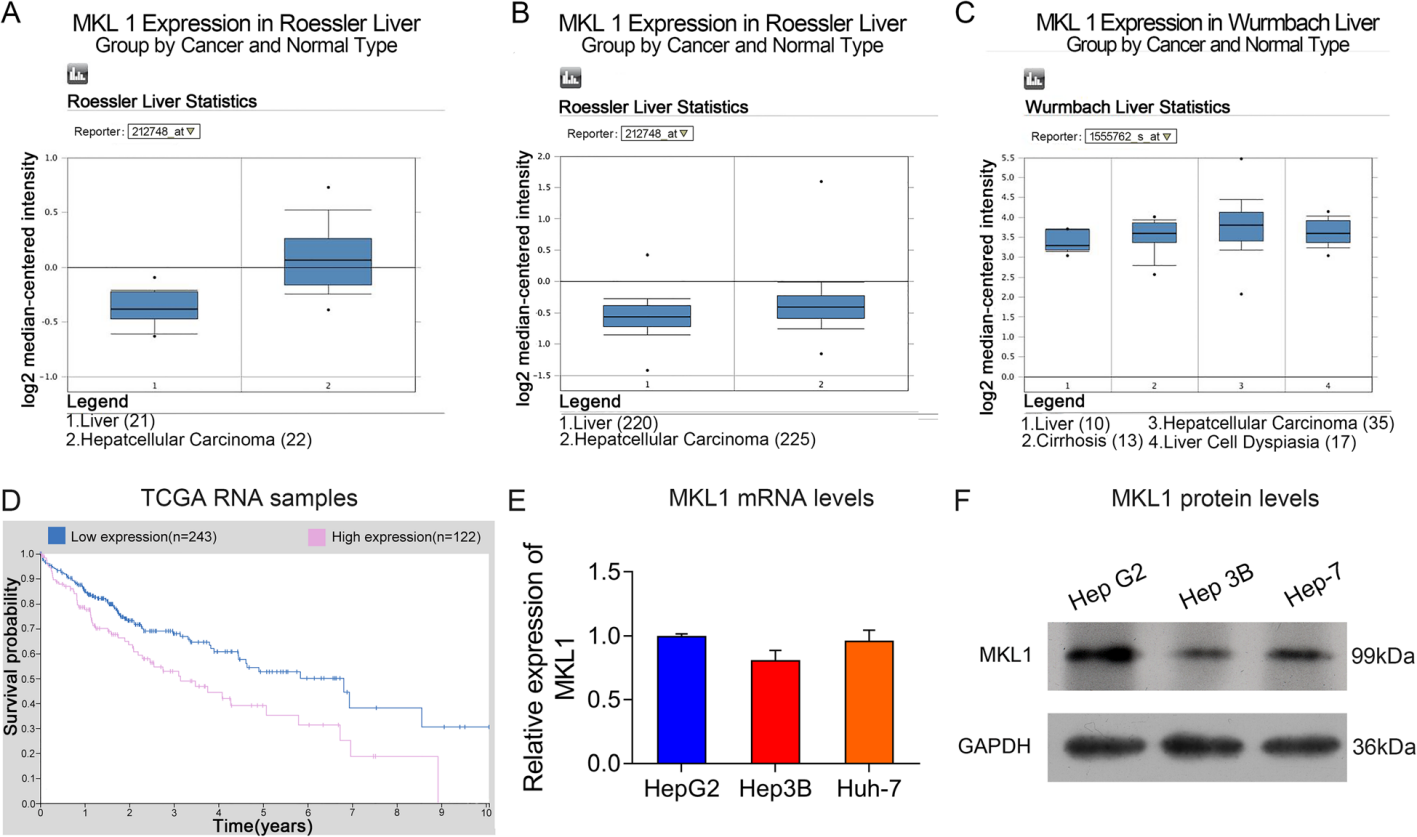
Elevated MKL1 gene expression in HCC tissues and cell lines. **a**-**b** The expressional differences of MKL1 gene between normal liver tissues and human hepatocellular carcinoma tissues. MKL1 gene expression was assessed by searching the Oncomine database. **c** The expression of MKL1 genes in normal liver tissues and liver tissues collected from patients with liver cirrhosis, hepatocellular carcinoma and liver cell dysplasia. MKL1 gene expression levels were evaluated using the Oncomine database. **d**. The correlation of MKL1 gene expression and survival of patients with HCC. The survivals of HCC patients with low or high expression of MKL1 gene were shown in blue and red lines respectively. **e** The MKL1 mRNA levels in hepatocellular carcinoma cell lines. MLK1 mRNA levels were detected by quantitative RT-PCR method. **f** Alterations of the MKL1 protein abundances in hepatocellular carcinoma cell lines. Western blotting was performed to detect MKL1 protein levels using GAPDH as the internal standard. Full-length blots/gels are presented in Additional files 1A. *P < 0.05; **P < 0.01

To explore the potential roles of MKL1 gene in HCC pathogenesis, we first analyzed the expression of MKL1 gene in both clinical tissues and cultured hepatocellular carcinoma cell lines. By searching against the Oncomine database, we showed that the expression of MKL1 gene in cancerous tissues collected from HCC patients were significantly elevated compared with the normal human liver tissues (Fig. 1a and b). Also, the Oncomine database showed that the expression of MKL1 gene in HCC patients were even higher than those patients diagnosed with liver cirrhosis and liver cell dysplasia, which were all significantly higher than normal human live tissues (Fig. 1c). Moreover, low expression of MLK1 gene was correlated with high survival of HCC patients (Fig. 1d). For validation of the high expression of MKL1 gene associated with HCC development, we then tested the expression of MKL1 gene in several hepatocellular carcinoma cell lines HepG2, Hep 3B and Huh-7. We observed by quantitative RT-PCR method that the MKL1 mRNA levels in these HCC cell lines (Fig. 1e). In consistence, the MLK1 protein abundances in these HCC cell lines (Fig. 1f). The HepG2 cells showed the highest expression of MKL1 gene among all these three HCC cell lines (Fig. 1f). The highly elevation of MKL1 gene in the clinical tissues and cell lines suggested its potential functions in HCC pathogenesis.

### MKL1 knockdown suppressed proliferation, migration and invasion but promoted apoptosis of HCC cells

**Fig. 2:**
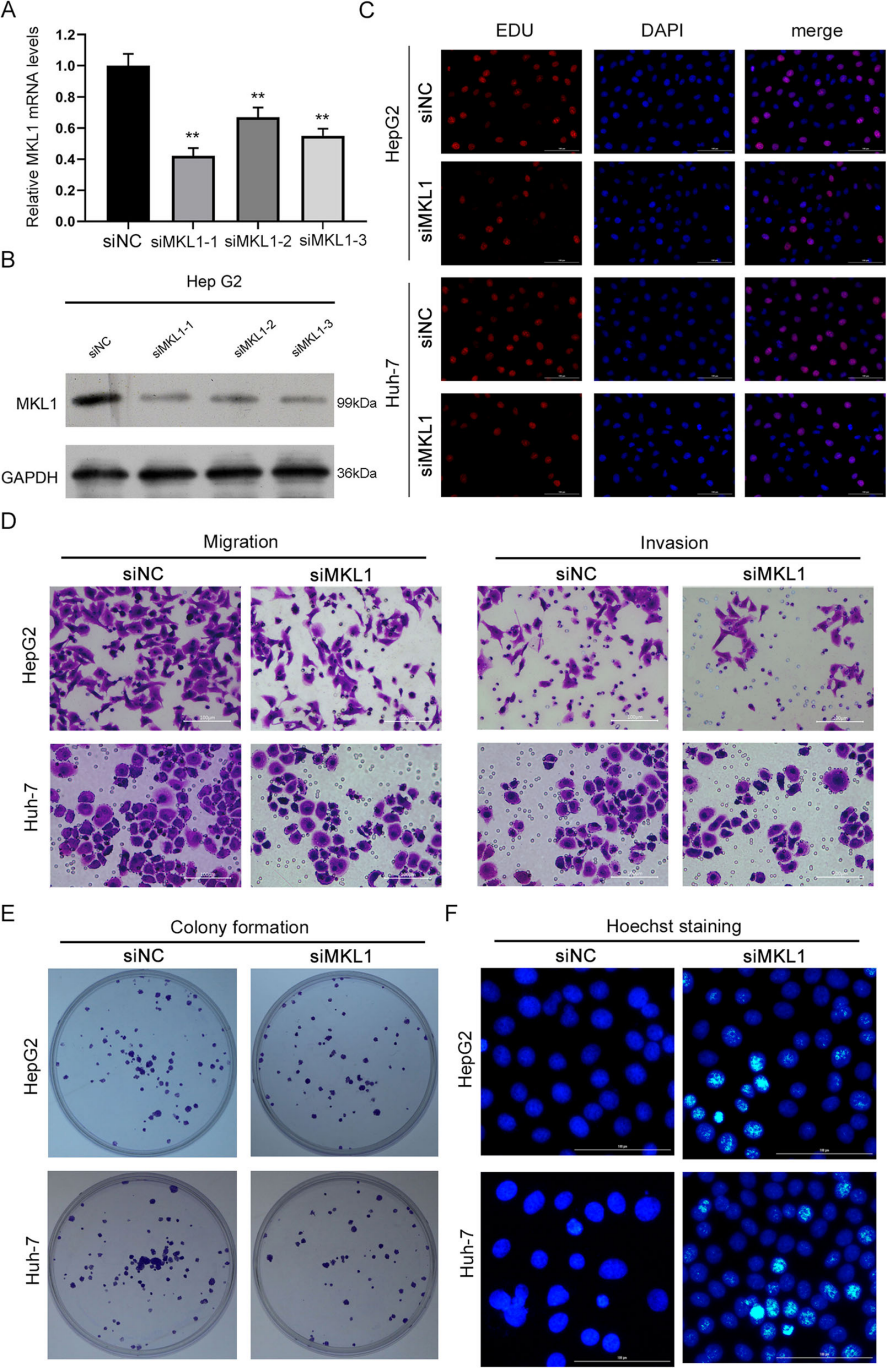
Modulation of HCC proliferation, migration, invasion and apoptosis by MKL silencing. **a**-**b** The knockdown of MKL1 gene expression in HepG2 cells by transfection with siMKL1 sequences. MKL1 mRNA and protein levels were detected by quantitative RT-PCR (**a**) and western blotting (**b**) respectively. **c** Inhibition of HepG2 and Huh-7 cell proliferation by silencing of MKL1 gene expression. Cell proliferation was analyzed by the Edu staining. Bar: 50 μm. **d** Suppression of HepG2 and Huh-7 cell migration and invasion induced by MKL1 gene silencing. The Transwell system was used to determine cell migration and invasion capacities. Bar: 20 μm. **e** The colony formation capacities of HepG2 and Huh-7 cells repressed by MKL1 gene silencing. Bar: 1.5 cm. **f** Promotion of HepG2 and Huh-7 cell apoptosis by silencing of MKL1 gene expression. Cell apoptosis was assessed by Hoechst staining. Bar: 20 μm. The bar graphs showing the effects in Figs. 2d-f are presented in Additional files 4. full-length blots/gels are presented in Additional files 1B MKL1: Megakaryoblastic leukemia 1; siMKL1: siRNAs targeting MKL1; NC: negative control; **P < 0.01

For knowledge of the pathogenic roles of MKL1 in HCC, we then tried the silencing of MKL1 gene expression in HepG2 and Huh-7 cells, which exhibited the highest MKL1 expression among these cell lines as shown in Fig. 1. We found that the transfection of the siRNAs targeting MKL1 all resulted into remarkable decreases of MKL1 mRNA and protein levels in in HepG2 and Huh-7 cells, compared with the NC group (Fig. 2a and b, Additional files 2). Among them, the siMKL1-I sequence caused even greater suppression of MKL1 gene expression in HepG2 cells, compared with other two siRNAs (Fig. 2a and b, Additional files 2). Subsequently, the siMKL1-I sequence was used to establish HepG2 and Huh-7 cells. Through the Edu staining, we showed that knockdown of MKL1 gene expression by siMKL1 caused significant repression of HepG2 and HuH-7 cell proliferation, compared with the siNC groups (Fig. 2c). Also, we found both the migration and invasion capacities of the HepG2 and Huh-7 cells were significantly suppressed by the silencing of MKL1 gene expression using siRNA sequences (Fig. 2d, Additional files 4). In consistence, the colony formation capacity of both the HepG2 and HuH-7 cells were also greatly repressed by siMKL1, comparted with the siNC group (Fig. 2e, Additional files 4). On the contrary, the apoptosis of HepG2 and HuH-7 cells were effectively promoted by transfection with siMKL1, in comparison with the siNC group (Fig. 2f, Additional files 4). In addition, inhibition of MKL1 significantly promoted the expression of Bax and cleaved-caspase3 proteins in HepG2 and Huh-7 cells, and inhibited the expression of Bcl-2 protein (Additional files 3). These results showed that the expression of MKL1 gene modulate the proliferation, migration, invasion and apoptosis of HCC cells.

### MKL1 promote HCC tumorigenesis by enhancing the COMPASS/NF-kB signaling

**Fig. 3:**
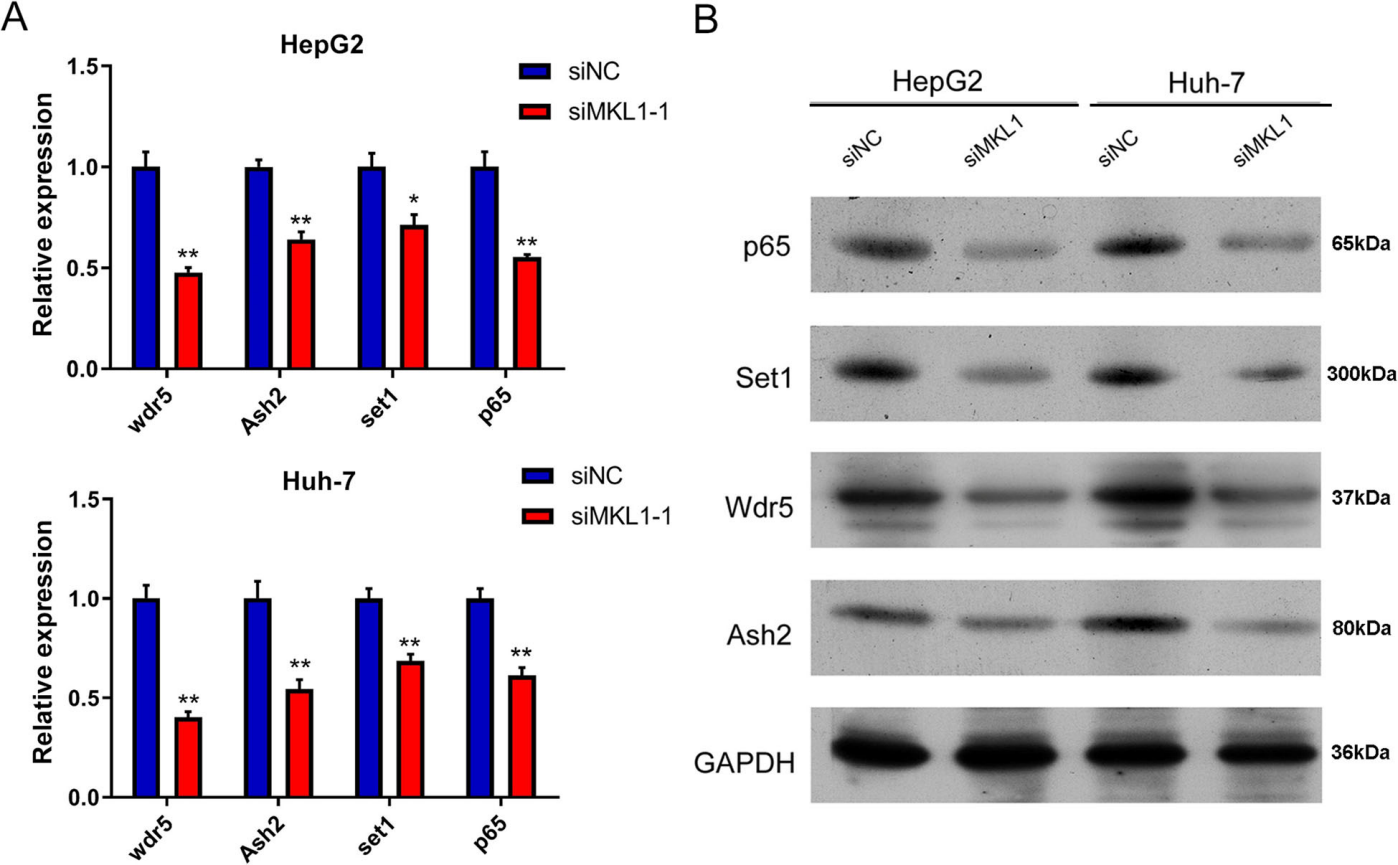
Inhibition of COMPASS complex and NF-kB signaling by MKL1 silencing in HCC cell lines. **a** The decreases of WRR5, Ash2, SET1 and p65 mRNA levels in HepG2 and Huh-7 cells induced by MKL1 silencing. The mRNA levels of WRR5, Ash2, SET1 and p65 genes were measured by quantitative RT-PCR method, using GAPDH as the internal standard. **b** The reduced abundances of WRR5, Ash2, SET1 and p65 proteins in HepG2 and Huh-7 cells transfected with siMKL1. WRR5, Ash2, SET1 and p65 protein levels in HCC cells were detected by western blotting, with GAPDH as the internal standard. Full-length blots/gels are presented in Additional files 1C *P < 0.05; **P < 0.01

For more insights into the signaling mechanism downstream of MKL1 in HCC pathogenesis, we then analyzed the alterations of the COMPASS complex and the NF-kB signaling in hepatocellular carcinoma cells induced by MKL1 gene silencing. Through quantitative RT-PCR method, we observed that the expression of WRR5, Ash2, SET1 and p65 genes in HepG2 cells were greatly suppressed by silencing of MKL1 gene expression, compared with those cells transfected with the negative control sequence (Fig. 3a). The repression of WRR5, Ash2, SET1 and p65 gene mRNA levels by silencing of MKL1 gene expression was also validated by quantitative RT-PCR in Huh-7 cells (Fig. 3a). In accordance, we showed that the protein levels of p65 in both the HepG2 and Huh-7 cells were significantly reduced by transfection with the siMKL1 sequences, compared with the negative control groups (Fig. 3b). Similarly, the abundances of WRR5, Ash2, SET1 proteins in both the HepG2 and Huh-7 cells transfected with siMKL1 were all markedly lower than those transfected with negative control sequences (Fig. 3b). In addition, we observed the expression of compass complex protein and MKL1 protein by knockdown p65 cells. The results showed that inhibition of p65 significantly inhibited the expression of MKL1, p65, WRR5, Ash2 and SET1 proteins, while further overexpression of MKL1 significantly increased the expression of these proteins (Additional files 5), which proved that MKL1 promoted the development of hepatocarcinoma cells by regulating the expression of COMPASS/NF-kB protein. These results demonstrated significant alteration of the COMPASS complex and NF-kB signaling pathways caused by MKL1 silencing in hepatocellular carcinoma cells, which indicted the mediating roles of the COMPASS/NF-kB axis in HCC pathogenesis promoted by MKL1.

### MKL1 knockdown impaired the tumorigenesis of HCC cells

**Fig. 4:**
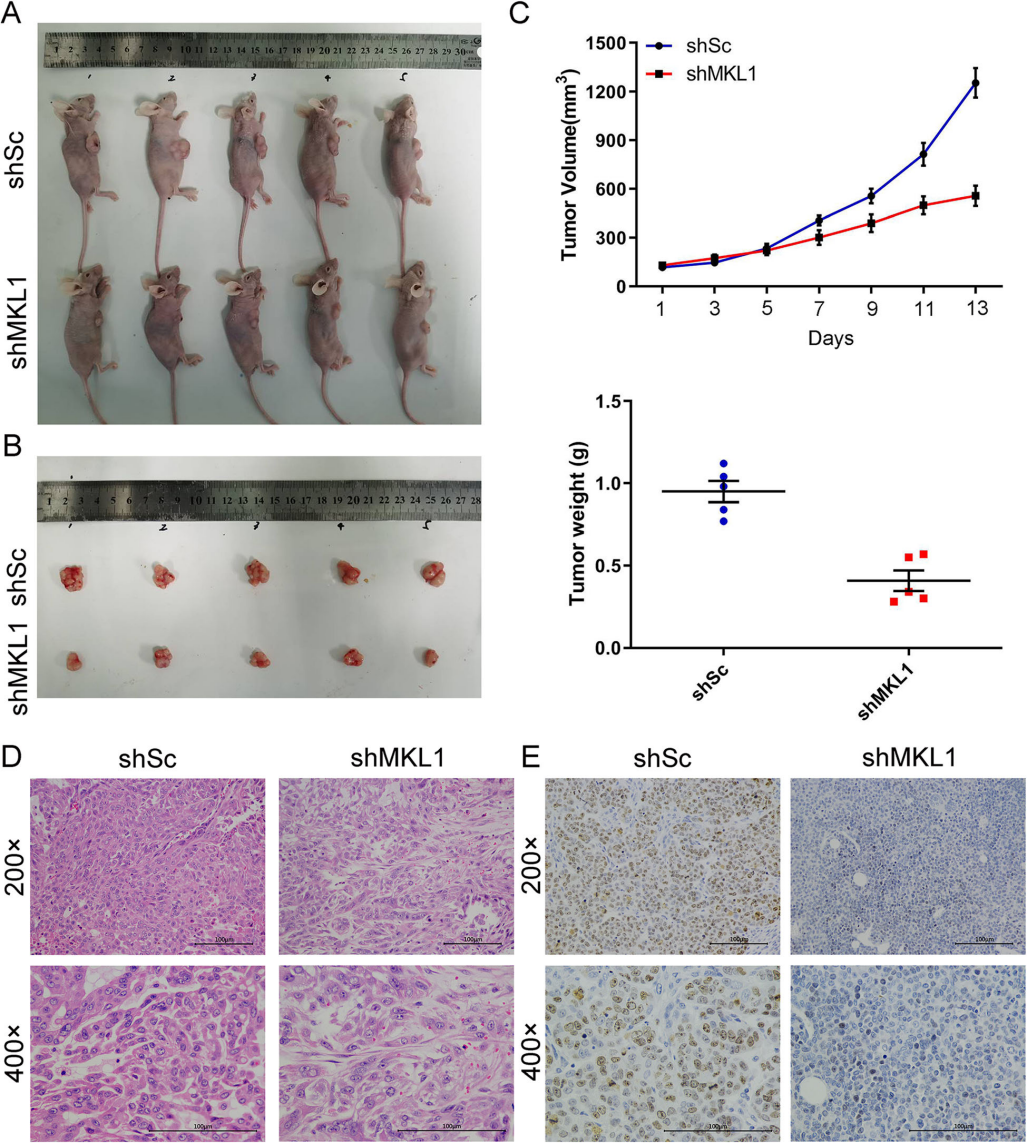
Impairment of HCC tumorigenesis by MKL1 knockdown in nude mice. **a** The cancer cell line-based xenograft (CDX) model used for evaluation of HCC cell tumorigenesis alteration induced by MKL2 knockdown. BALB/c nude mice were subcutaneously injected with HepG2 cells transfected with shMKL1 or shSC. **b** The tumors formed in nude mice injected with HepG2 cells transfected with shMKL1 or shSC. **c** Quantitation of the tumors formed in nude mice induced by injection of HepG2 cells with or without MKL1 knockdown. The volumes of tumors were recorded for successive 13 days (**c**) and the tumor wet weights were measured at the 13th day when mice were sacrificed. **d** The histological alterations of tumors induced by MKL1 knockdown HepG2 cells in nude mice. Histological features of tumors were analyzed following H&E staining. **e** Reduced Ki67 protein levels in the tumors induced by MKL1- knockdown HepG2 cells in nude mice. Ki67 expression in tumor tissues were analyzed by the IHC assay. **P < 0.01

To analyze the influences of MKL1 gene expression on the tumorigenesis of hepatocellular carcinoma cells, we established the cancer cell line-based xenograft (CDX) model to evaluate the alteration of in vivo tumorigenesis induced by MKL1 gene knockdown (Fig. 4a and b). In this model, we observed that the volumes of tumors generated in nude mice injected with the HepG2 cells transfected with MKL1 shRNAs were significantly smaller than those induced by normal HepG2 cells, especially since the 9th day after cancer cell injection (Fig. 4b and c). Also, the wet weights of tumors induced by HepG2 cells with MKL1 gene knockdown in nude mice were also remarkably lowered, compared with those formed from normal HepG2 cells (Fig. 4c). Moreover, our H&E staining revealed much decreased cell density and sparse cell lining in the tumors generated in nude mice injected with HepG2 cells with MKL1 silencing, in contrast to those induced by normal HepG2 cells (Fig. 4d). In addition, our IHC assay using antibodies targeting Ki67 proteins showed significantly lower expression of Ki67 proteins in the tumors induced by MKL1knockdown HepG2 cells, compared with those induced by normal HepG2 cells (Fig. 4e). Together, these results showed that the knockdown of MKL1 gene expression repressed the proliferation rates and in vivo tumorigenesis of hepatocellular carcinoma cells.

## Discussion

The alteration of epigenetic machineries such as histone methylations has been identified as essential mechanisms driving initiation and progression of human cancers including the hepatocellular carcinoma [18, 20]. As one major types of histone modification associated with epigenetic events, the COMPASS complex is responsible for catalyzing the trimethylation of H3K4 and target gene transcription associated with the prognosis of hepatocellular carcinoma patients [21, 22]. However, the functional regulation of the COMPASS complex and downstream signaling pathways during HCC pathogenesis remains poorly elucidated. In the present study, we first demonstrated the highly elevated expression of MKL1 gene in the clinical tissues from HCC patients by bioinformatic analysis, combined with validation in three HCC cell lines. Moreover, the silencing of MKL1 expression resulted into significantly repressed proliferation, migration, invasion and colony formation but promoted apoptosis in HCC cells. In addition, we confirmed by cancer cell line-based xenograft model that MKL1 gene silencing impaired the tumorigenesis of HCC cells. Finally, we showed that MKL1 silencing caused greatly reduced expression of three COMPASS complex components and repressed downstream NF-kF signaling in HCC cells. These results provided novels insights into the molecular mechanism underlying HCC development, especially the regulation of epigenetic events mediated by histone lysine methylation.

MKL1 (Megakaryoblastic leukemia 1) was previously identified as one part of translocation mutation associated with acute megakaryoblastic leukemia, which is one member of the myocardin transcriptional coactivator family featured with several conserved domains such as C-terminal transactivation domain, a leucine zipper-like domain and a glutamine-rich domain [26]. Reports in recent years showed that MKL1 has been widely implicated in various biological and pathogenic processes such as cell migration, chromatin reorganization, histone post-translational modification, dendritic spine morphology, angiogenesis, immune responses and also cancer development [27–30]. For instance, MKL1 could be recruited onto the promoter of the matrix metalloproteinase 2 (MMP2) gene in ovarian cancer cells as a response to hypoxia, and promote MMP2 gene transcription and ovarian cancer cell migration and invasion by recruiting the SET1 and BRG1, which serve as histone methyltransferase and chromatin remodeling protein respectively [31]. Also, MKL1 was involved in endothelial-to-mesenchymal transition that mediated the liver fibrosis and cirrhosis [23]. Moreover, the depletion of MKL1 gene effectively repressed the growth of hepatocellular carcinoma xenograft through enhancing the oncogene-induced senescence [24]. In the present study, we demonstrated the significantly increased MKL1 expression in HCC clinical tissues and cell lines, followed by direct evidences showing the regulation of HCC proliferation, migration, invasion, apoptosis and in vivo tumorigenesis by MKL1 silencing. The regulation of HCC proliferation by MKL1 was also validated by the reduced Ki67 expression in tumors caused by MKL1-silenced HCC cells in nude mice. Our investigation further validated the expressional alterations and roles of MKL1 in HCC pathogenesis, which also provided further insights into the cellular functions of MKL1 high expression in HCC initiation and progression.

As introduced above, the modulation of histone methylation and chromatin remodeling has been shown as one key mechanism of MKL1-regulated target gene transcription activation [21, 31]. Previous investigations showed that the transcription-enhancing functions of MKL1 protein was mainly mediated by the recruitment of the COMPASS complex and resultant histone lysine methylation [21, 31]. The COMPASS complex is composed of three major protein components, including SET1 protein responsible for catalyzing the lysine methylation in histones wrapped by target gene sequences, and WDR5 and ASH2 serving as histone methyltransferase adaptors [21, 32, 33]. In this study, we showed that the expression of SET1, WDR5 and ASH2 proteins in HepG2 and Huh-7 cells were all significantly down-regulated by silencing of MKL1 gene expression, which convincingly verified the effects of MKL1 protein in regulating the COMPASS complex functions in context of hepatocellular carcinoma. Interesting, the regulation of SET1, WDR5 and ASH2 proteins by MKL1 was also recently observed in hepatic stellate cells during TGF-(tumor growth factor b)-induced fibrogenic response and fibrosis in the liver [34]. Combined together, it is reasonably to conclude that the MKL1-regulated COMPASS complex serves as an essential diver of HCC pathogenesis, through promoting liver fibrosis and modulating hepatocellular carcinoma cell proliferation, migration, invasion and apoptosis.

The regulation of target gene transcription by MKL1 and the COMPASS complex has also been regulated by their reciprocal interactions with the NF-kB signaling pathway. On one hand, the NF-κB protein was required for the association of MKL1 protein with the promoter regions of target genes, thus initiating the following COMPASS complex-induced histone modification and transcription activation [21, 31]. On the other hand, the introduction of MKL1 protein could also enhance the nuclear enrichment of the NF-kB protein and strength the binding of NF-kB protein with target gene promoter, therefore promoting further elevated expression of NF-kB target genes [21]. Here in this study, we also showed that the expression of p65 protein, one major subunit of the NF-kB protein, was remarkably decreased in both the HepG2 and Huh-7 cells, indicating the involvement of NF-kB signaling and its interaction with the COMPASS complex during MKL1-regulated HCC pathogenesis. Of note, the dynamic alteration and molecular mechanisms underlying the interaction between MKL1 protein, the COMPASS complex and NF-kB in HCC cells deserve further investigation.

## Conclusions

In summary, we unveiled in this study that the high expression of MKL1 protein could promote HCC pathogenesis by enhancing hepatic cell proliferation, migration and invasion and inhibiting liver cell apoptosis, which was mediated by regulating the COMPASS complex and NF-kB signaling pathway. This new MKL1/COMPASS/NF-kB axis in HCC pathogenesis could be further explored as targets for HCC diagnosis and treatment.

## Supplementary Information

**Additional file 1:** The original bands of WB.

**Additional file 2:** MKL1 protein expression in Huh-7 cells with siMKL1.

**Additional file 3:** The apoptosis-related protein abundances in HepG2 and Huh-7 with siMKL1.

**Additional file 4:** The bar graphs show effect in Figs. 2d-f.

**Additional file 5:** The expression of COMPASS complex protein and MKL1 in HepG2 transfection with siP65.

## Data Availability

The datasets used or analyzed during the present study are available from the first author or corresponding author upon reasonable request.

## Abbreviations

COMPASS: Complex of proteins associated with Set1
HCC: Hepatocellular carcinoma
MLK1: Megakaryoblastic leukemia 1
NAFLD: Nonalcoholic fatty liver diseases
NC: Negative control
NASH: Nonalcoholic steatohepatitis
Sc: Scramble
