# Supplementary figures and images for: MKL1 regulates hepatocellular carcinoma cell proliferation, migration and apoptosis via the COMPASS complex and NF-κB signaling
Source: BMC Cancer. 2021 Nov 6;21:1184. doi: 10.1186/s12885-021-08185-w (PMC8571910; doi:10.1186/s12885-021-08185-w)

The original bands of WB

Figure A：


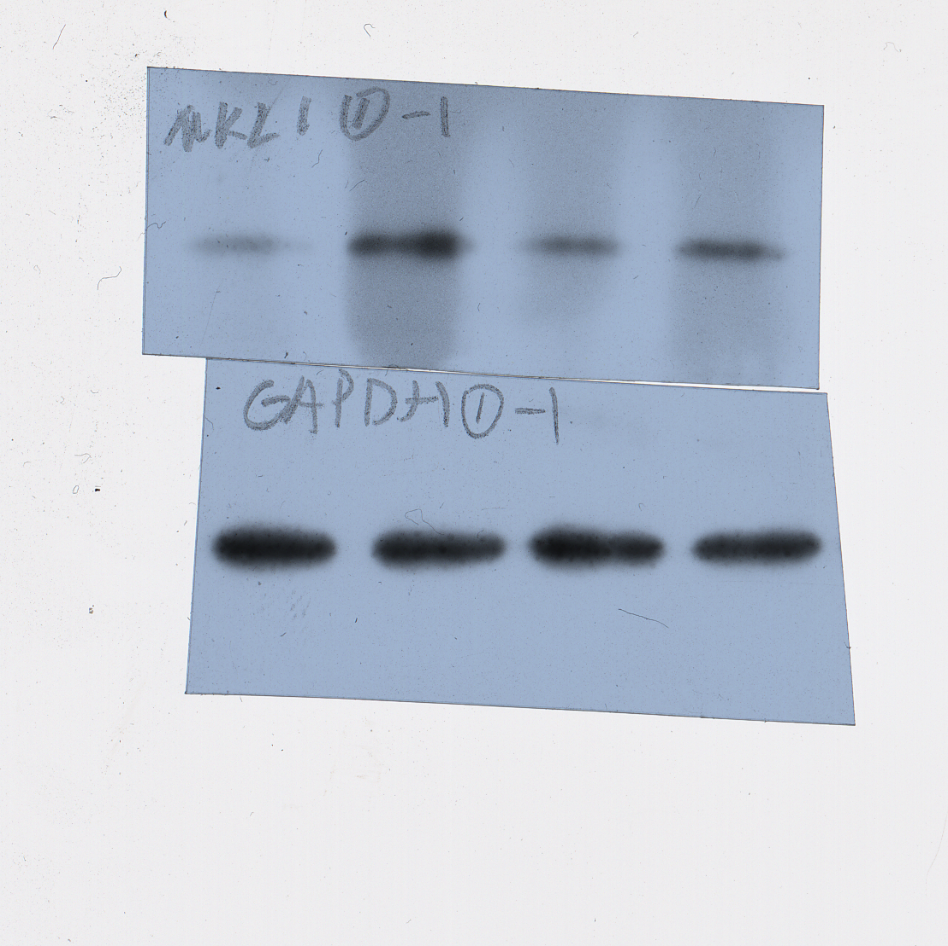


Figure B：


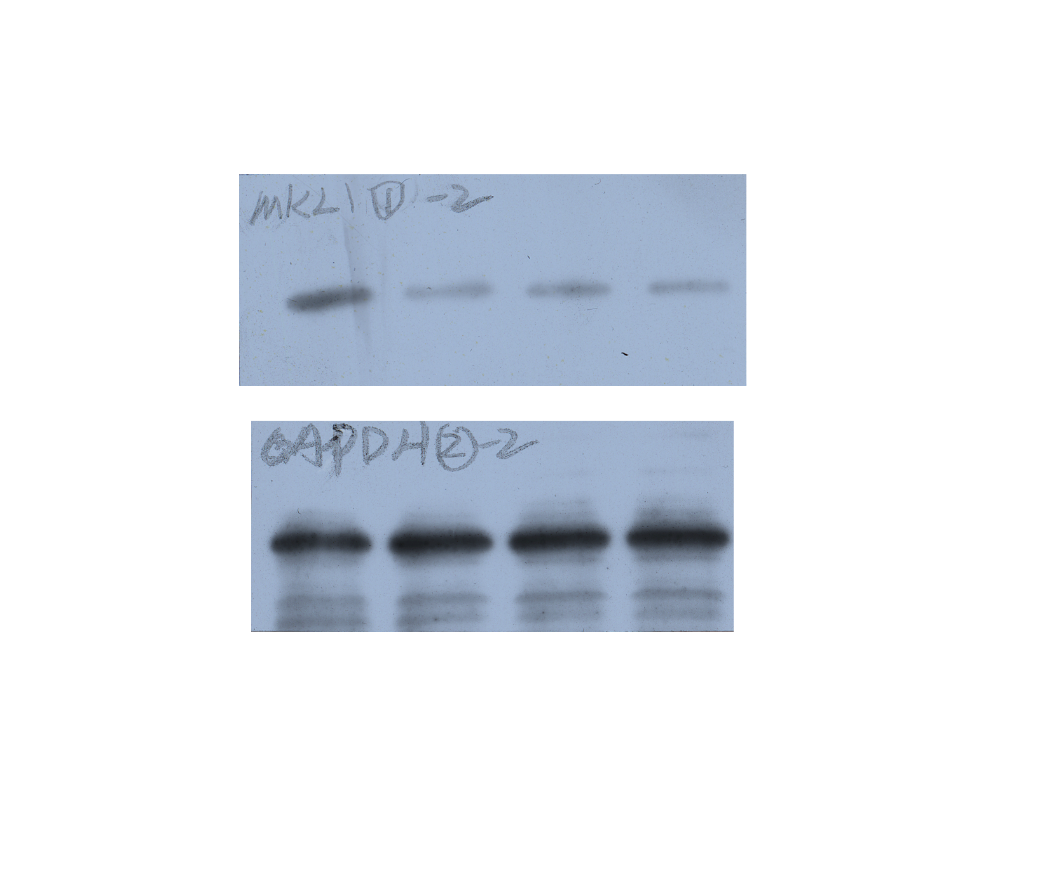


Figure C：


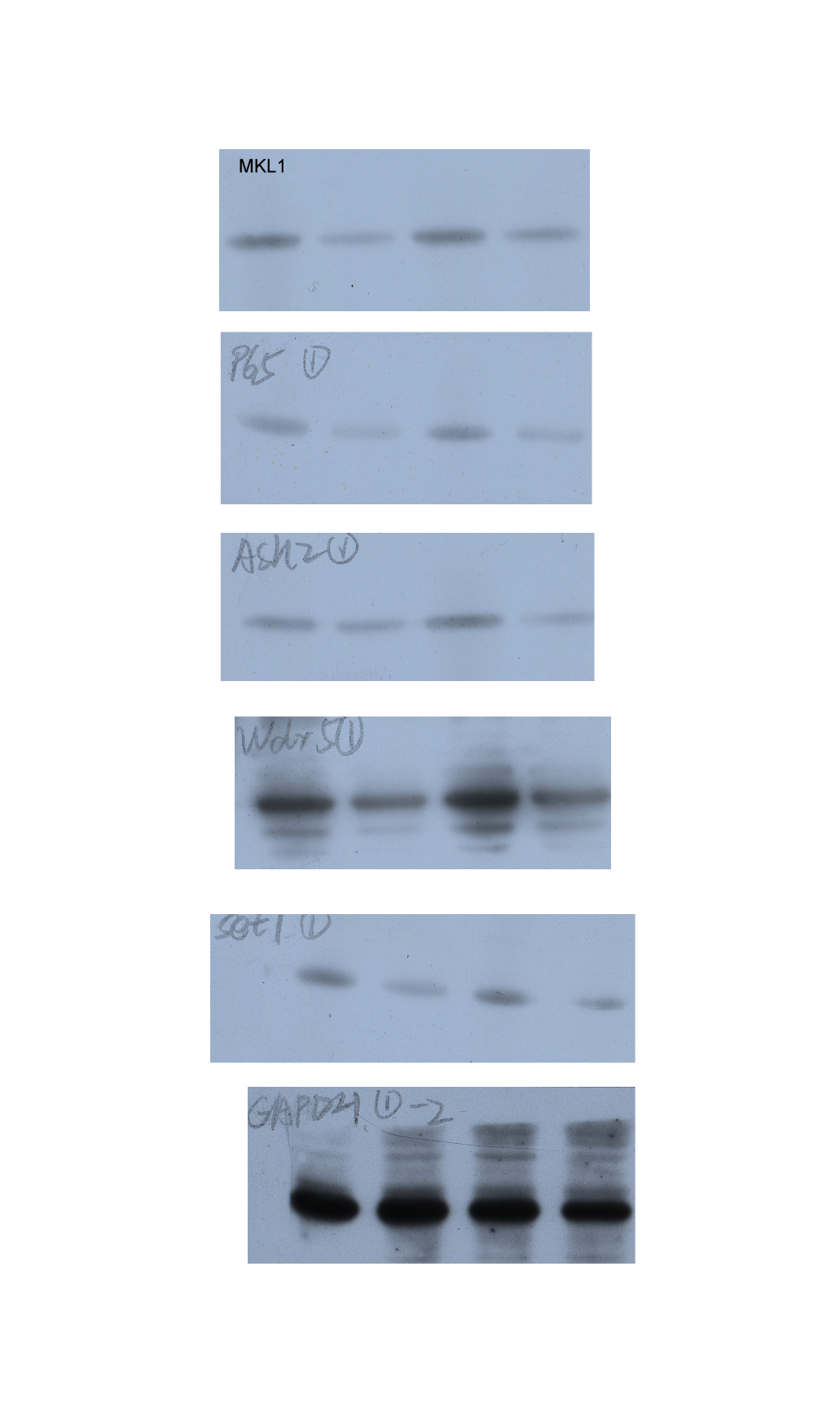

Supplement: Supplementary file 1 — Additional file 1: The original bands of WB. [file 12885_2021_8185_MOESM1_ESM.docx]
