# Supplementary material for: MKL1 regulates hepatocellular carcinoma cell proliferation, migration and apoptosis via the COMPASS complex and NF-κB signaling
Source: BMC Cancer. 2021 Nov 6;21:1184. doi: 10.1186/s12885-021-08185-w (PMC8571910; doi:10.1186/s12885-021-08185-w)
Supplement: Supplementary file 2 — Additional file 2: MKL1 protein expression in Huh-7 cells with siMKL1. [file 12885_2021_8185_MOESM2_ESM.docx]

**MKL1 protein expression in Huh-7 cells with siMKL1**


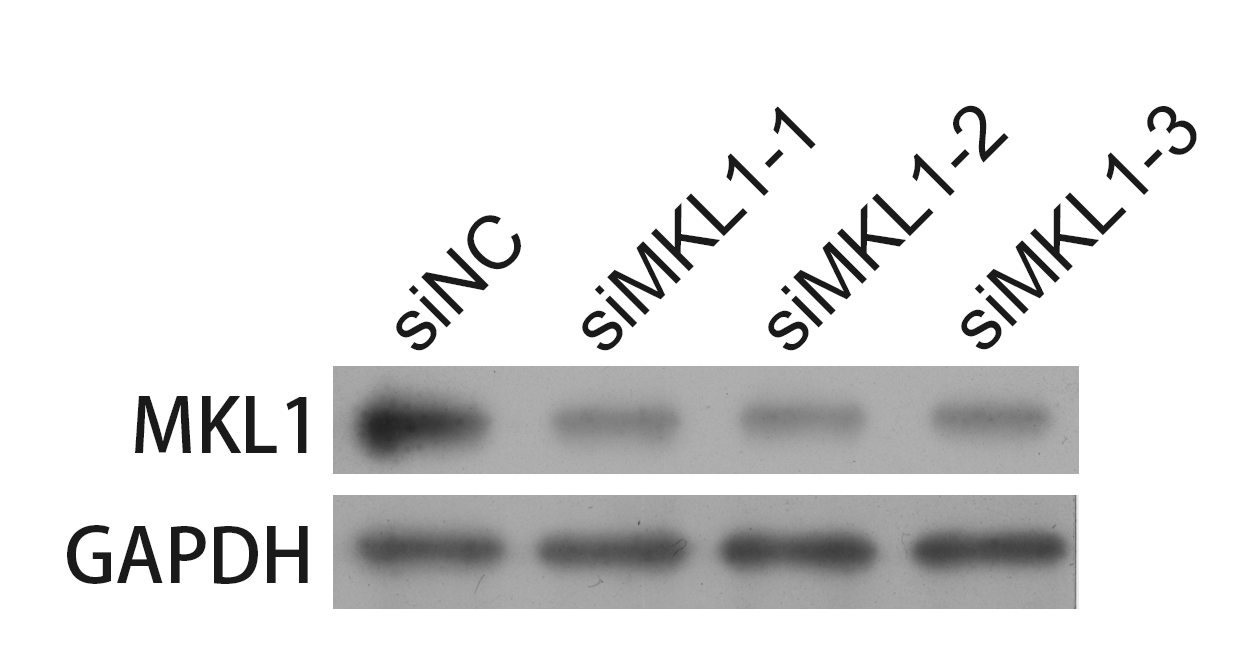


**Supplementary Figure 1**

**Figure ledgend：**

The knockdown of MKL1 gene expression in Huh-7 cells by transfection with siMKL1 sequences. MKL1 protein levels were detected by western blotting with GAPDH as the internal standard.
