# Supplementary material for: MKL1 regulates hepatocellular carcinoma cell proliferation, migration and apoptosis via the COMPASS complex and NF-κB signaling
Source: BMC Cancer. 2021 Nov 6;21:1184. doi: 10.1186/s12885-021-08185-w (PMC8571910; doi:10.1186/s12885-021-08185-w)
Supplement: Supplementary file 3 — Additional file 3: The apoptosis-related protein abundances in HepG2 and Huh-7 with siMKL1. [file 12885_2021_8185_MOESM3_ESM.docx]

**The apoptosis-related protein abundances in HepG2 and Huh-7 with siMKL1**
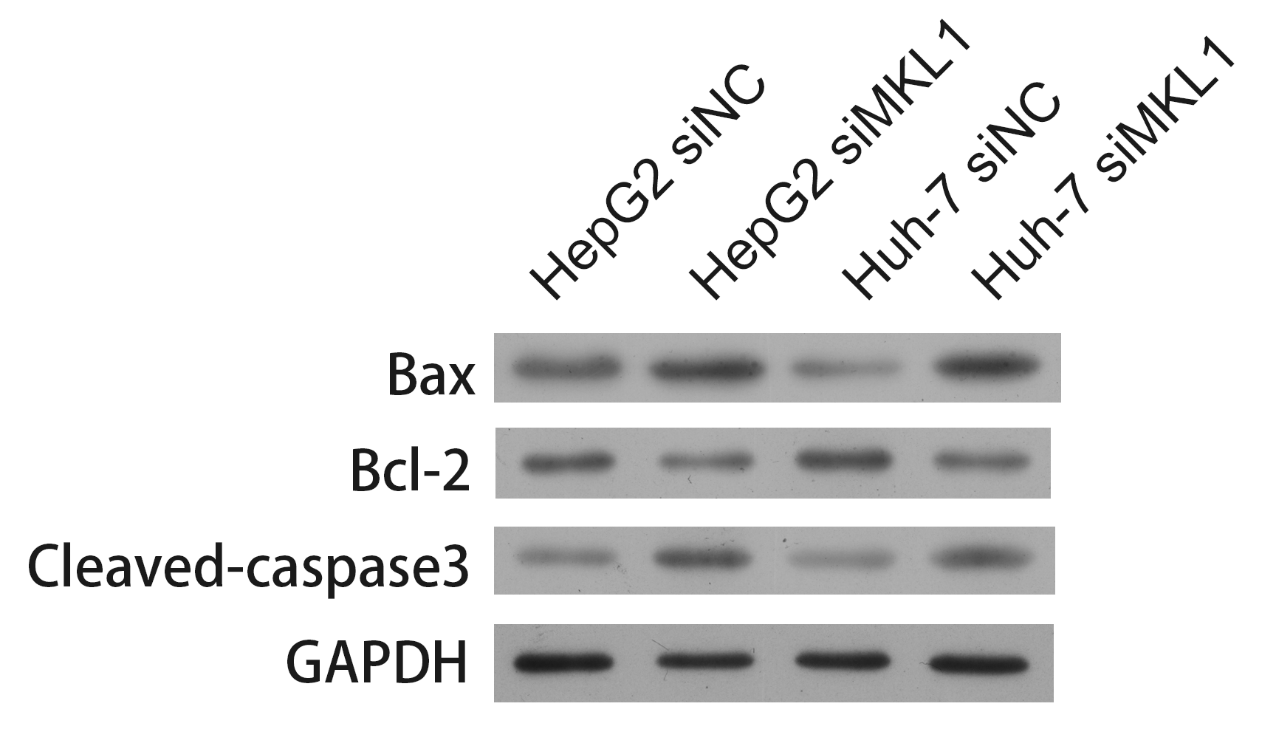


**Supplementary Figure 2**

**Figure ledgend：**

Suppression of HepG2 and Huh-7 the protein expression of Bax, Bcl-2 and cleased-caspase3 induced by MKL1 gene silencing. Bax, Bcl-2 and cleased-caspase3 protein levels in HCC cells were detected by western blotting, with GAPDH as the internal standard.
