# Supplementary material for: MKL1 regulates hepatocellular carcinoma cell proliferation, migration and apoptosis via the COMPASS complex and NF-κB signaling
Source: BMC Cancer. 2021 Nov 6;21:1184. doi: 10.1186/s12885-021-08185-w (PMC8571910; doi:10.1186/s12885-021-08185-w)
Supplement: Supplementary file 4 — Additional file 4: The bar graphs show effect in Figs. 2d-f. [file 12885_2021_8185_MOESM4_ESM.docx]

**The bar graphs show effect in Figures 2D-F：**


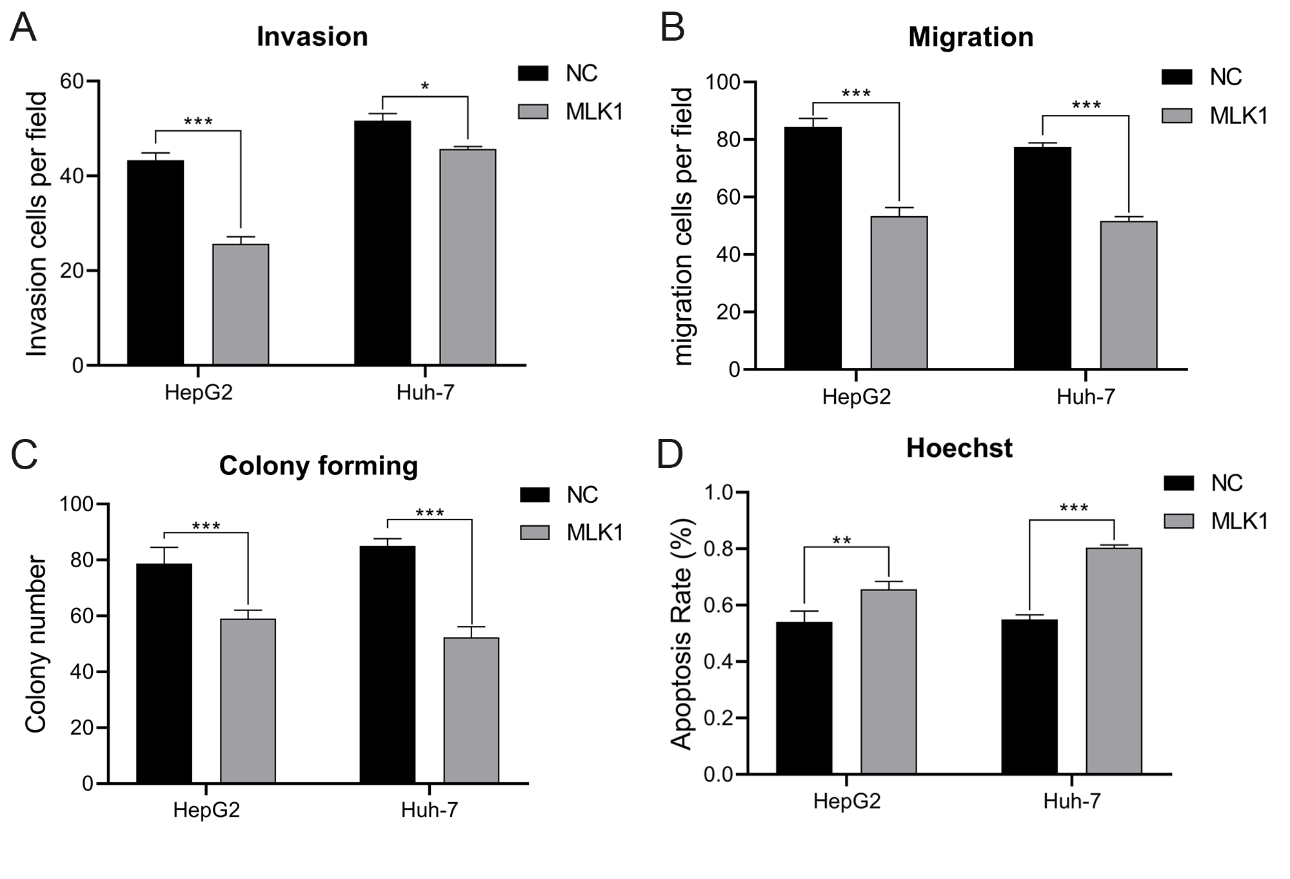


**Supplementary Figure 3**

**Figure ledgend：**

Suppression of HepG2 and Huh-7 cell migration, invasion, colony formation capacities and apoptosis induced by MKL1 gene silencing. *P<0.05; **P<0.01.
