# Supplementary material for: MKL1 regulates hepatocellular carcinoma cell proliferation, migration and apoptosis via the COMPASS complex and NF-κB signaling
Source: BMC Cancer. 2021 Nov 6;21:1184. doi: 10.1186/s12885-021-08185-w (PMC8571910; doi:10.1186/s12885-021-08185-w)
Supplement: Supplementary file 5 — Additional file 5: The expression of COMPASS complex protein and MKL1 in HepG2 transfection with siP65. [file 12885_2021_8185_MOESM5_ESM.docx]

**The** **expression of COMPASS complex protein and MKL1 in HepG2 transfection with siP65：**


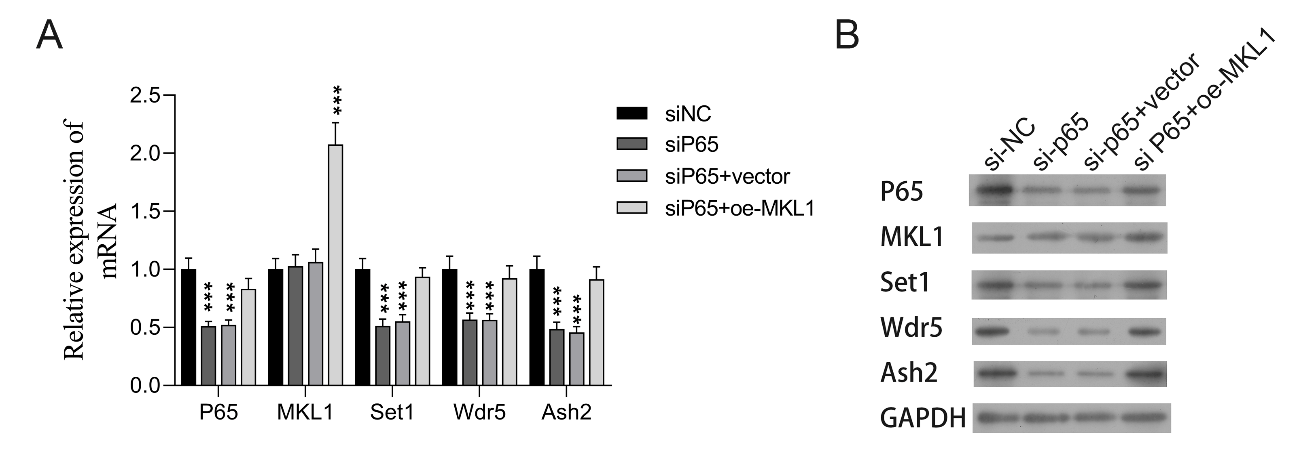


**Supplementary Figure 4**

**Figure ledgend**

(A) The mRNA expression of COMPASS complex protein and MKL1 protein were detected by transfection of siP65. (B) The protein expression of COMPASS complex protein and MKL1 protein were detected by transfection of siP65. MKL1, WRR5, Ash2, SET1 and p65 protein levels in HCC cells were detected by western blotting, with GAPDH as the internal standard. *P<0.05; **P<0.01.
